# Multiple sclerosis patients’ response to COVID-19 pandemic and vaccination: correspondence

**DOI:** 10.1186/s41983-023-00615-9

**Published:** 2023-01-26

**Authors:** Rujittika Mungmunpuntipantip, Viroj Wiwanitkit

**Affiliations:** 1 Private Academic Consultant, Bangkok, Thailand; 2 Joseph Ayobabalola University, Ikeji-Arakeji, Nigeria; 3 Dr. D. Y. Patil Medical College, Dr. D. Y. Patil Vidyapeeth, Pune, India

Dear Editor,

We would like to discuss “Multiple sclerosis patients’ response to COVID-19 pandemic and vaccination in Egypt [1].” Gad et al. came to the conclusion that the risk of relapse is minimal with either infection or immunization, and that the prevalence and severity of COVID-19 infection were comparable to those of the general population [1]. No serious adverse reactions were documented following vaccination, according to Gad et al. [1]. It is interesting to see how infections and COVID-19 vaccination affect people with multiple sclerosis. The current report may be helpful in patient vaccination planning. The recipient’s general health and whether they are undergoing treatment for an underlying illness may both affect how effective the COVID-19 immunization is. To get at the correct interpretation, a number of factors must be taken into account. A genuine bad reaction was one of the possible confounding factors that might have had an impact on the results of the initial dose. Examples include the COVID-19 strain, the delivery mechanism, the setting, and the recipient’s co-morbidity prior to vaccination. The absence of clinical symptoms and asymptomatic COVID-19 may be related [2]. It is necessary to rule out a COVID-19 with no prior or present symptoms. The chance of cross-contamination with an unidentified SARS-CoV-2 infection cannot be completely ruled out. Investigation and confirmation of the effect of inherited genetic variability on vaccine recipients’ immunological reaction have also been made [3]. More empirical investigation should be conducted to ascertain whether or not the current report is substantiated.

## Data Availability

There is no new data generated.

## Abbreviations

COVID-19: Coronavirus 2019
SARS-CoV-2: Severe acute respiratory syndrome coronavirus 2
